# CRISPR/Cas12a Collateral Cleavage Activity for Sensitive 3′–5′ Exonuclease Assay

**DOI:** 10.3390/bios13110963

**Published:** 2023-10-30

**Authors:** Jae Hoon Jeung, Hyogu Han, Chang Yeol Lee, Jun Ki Ahn

**Affiliations:** 1 Material & Component Convergence R&D Department, Korea Institute of Industrial Technology (KITECH), Ansan 15588, Republic of Korea; qaz2758@kitech.re.kr (J.H.J.); ninehyo@kitech.re.kr (H.H.); 2 Department of Biological Engineering, College of Engineering, Konkuk University, Seoul 05029, Republic of Korea; 3 Department of Chemistry, Gangneung–Wonju National University, Gangneung 25457, Republic of Korea; 4 Bionanotechnology Research Center, Korea Research Institute of Bioscience and Biotechnology (KRIBB), 125 Gwahak–ro, Yuseong–gu, Daejeon 34141, Republic of Korea

**Keywords:** Exonuclease III, CRISPR/Cas12a, hairpin probe, collateral cleavage, biosensor

## Abstract

This study presents a technique for detecting 3′–5′ exonuclease activity through the use of CRISPR/Cas12a. These enzymes, including 3′–5′ exonuclease (Exo III), perform crucial roles in various cellular processes and are associated with life expectancy. However, imbalances in their expression can increase susceptibility to diseases such as cancer, particularly under prolonged stress. In this study, an activator sequence of CRISPR/Cas12a was constructed on the 5′–end of a hairpin probe (HP), forming a blunt end. When the 3′–end of the HP was hydrolyzed with Exo III activity, the activator sequence of Cas12a was exposed, which led to collateral cleavage of the DNA signal probe and generated a fluorescent signal, allowing sensitive and highly specific Exo III detection. This detection principle relied on the fact that Exo III exclusively cleaves the 3′–end mononucleotide of dsDNA and does not affect ssDNA. Based on this strategy, Exo III activity was successfully assayed at 0.0073 U/mL, demonstrating high sensitivity. In addition, this technique was used to screen candidate inhibitors of Exo III activity.

## 1. Introduction

Exonucleases are a type of DNase that cleave the 3′–end or 5′–end of DNA by hydrolyzing phosphodiester bonds [1]. Exonuclease III (Exo III), which belongs to the exonuclease family, exhibits 3′–5′ exonuclease activity. It hydrolyzes the blunt 3′–end of dsDNA without recognizing a specific site but exhibits low activity against ssDNAs and dsDNAs with a 3′ overhang [2,3]. Previous studies have shown that 3′–5′ exonucleases perform several crucial roles in cellular and physiological processes [4]. Additionally, 3′–5′ exonucleases are associated with life expectancy and are specifically involved in stabilizing the mutation rate in human cells [5]. They also enable polymerase proofreading and increase the accuracy of DNA synthesis and replication [6]. However, overexpression or lack of 3′–5′exonucleases leads to increased vulnerability to diseases, including cancer, under long-term stress conditions [7]. Therefore, the validation of 3′–5′ exonuclease activity holds vital significance in the context of disease diagnosis, underscoring the need for a robust, efficient, and sensitive detection methodology that exhibits exceptional specificity for the identification of 3′–5′ exonucleases. Some of the common methods for detecting 3′–5′ exonuclease activity include radiolabeling and gel electrophoresis. However, these methods are time consuming, labor intensive, and present safety risks during detection [8,9,10].

In response to the limitations inherent in conventional methodologies, novel approaches for the detection of 3′–5′ exonuclease activity have emerged in recent times, aiming to address and overcome these shortcomings. For example, Gang et al., detected Exo III activity using DNA–templated Cu nanoclusters [11]. Su et al., verified the activity of Exo III using Cu nanoparticles and fluorescent probes [12]. Although both nanomaterial–based methods are highly sensitive, they are not suitable for rapid diagnosis due to the complexity and time–consuming nature of nanomaterial synthesis, as well as the requirement of a skilled workforce [13,14,15,16,17]. Furthermore, methods utilizing fluorescent signals to detect Exo III activity, while convenient, exhibit suboptimal sensitivity [18]. However, there is still potential for further improvement in their analytical performance. Therefore, it is valuable to develop advanced techniques for the 3′–5′ exonuclease assay that offers enhanced sensitivity, specificity, and/or convenience.

The clustered and regularly interspaced short palindromic repeats (CRISPRs)/Cas system is part of the immune system of prokaryotes, including archaea and bacteria, and is involved in antiviral activity. In this system, the genomic records of foreign invaders are stored in CRISPR arrays and placed between unique sequences called spacers. Subsequently, upon reinvasion by a foreign nucleic acid, the prokaryote recalls the previous invasion and produces Cas protein, forming a Cas protein/crRNA/DNA complex, which leads to DNA cleavage [19,20].

The revelation of the CRISPRs system’s existence had a profound impact on the field of genome editing technology, as evidenced by its transformative influence on the landscape of genetic manipulation [21], Furthermore, its rapid adoption for applications in molecular diagnosis further underscores the versatility and significance of the CRISPRs system [22]. Cas9 attaches to a matching DNA sequence through guide RNA (gRNA) molecules, known as specificity determinant RNA, and subsequently initiates cleavage of the corresponding DNA sequence using its nucleolytic capability referred to as cis–cleavage activity [23]. On the other hand, it has been discovered that in the case of Cas12a and Cas13a; they not only exhibit cis–cleavage but also collateral cleavage activity [24]. In the CRISPRs/Cas12a system, gRNA specifically recognizes dsDNA and ssDNA to form a complex. To bind to dsDNA, the protospacer adjacent motif (PAM) site, which has a TTTN sequence, is required [25]. In contrast, when a complex with ssDNA is formed, DNA cleavage can occur even without the PAM site [26]. After the Cas12a/gRNA/DNA complex is formed, it randomly cleaves nearby ssDNAs via collateral cleavage [24,26,27]. Similarly, Cas13a, after performing cis–cleavage on a ss RNA, undergoes a transformation into a nonspecific endonuclease with the ability to cleave nearby ss RNA sequences without discrimination [28]. Novel technologies have been developed recently, utilizing signal amplification through cleavage of nearby ssDNA reporters upon recognition of target by Cas12a and Cas13a. For instance, various nucleic acid detection methods have been developed, including the one–hour low–cost multipurpose highly efficient system (HOLMES) [29] and Specific High sensitivity Enzymatic Reporter unlocking (SHERLOCK) [28]. Furthermore, recently, advancements have been made to detect not only nucleic acids but also the activity of various enzymes and heavy metals using CRISPR–associated proteins [27,30,31,32]. However, despite these numerous advancements, progress in the rapid detection of non–nucleic acid substances such as enzyme activity lags behind that of nucleic acid detection using the CRISPRs/Cas system [33]. Thus, the quest for fresh approaches to convert molecular recognition occurrences into activators for CRISPRs–Cas12a is highly significant in extending the utility of the CRISPRs–Cas12a system and promoting its evolution.

**Figure 1:**
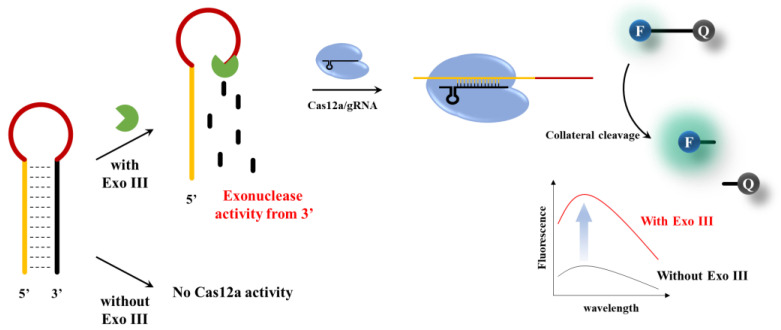
Schematic illustration of the Exo III activity detection based on CRISPR/Cas12a collateral cleavage activity.

In this study, we have developed an efficient, highly specific, and remarkably sensitive technique for detecting 3′–5′ exonuclease activity using the CRISPRs/Cas12a platform. As shown in Figure 1, the sequence of the Activator, which is complementary to the gRNA, spans from the 5′ end to the loop of the hairpin probe (HP). When the 3′–end of the HP is hydrolyzed by exonuclease activity, The Activator sequence becomes exposed, enabling it to bind with the Cas12a/gRNA complex. This leads to the collateral cleavage of the DNA signal probe, generating a fluorescent signal. This allows sensitive and highly specific detection of 3′–5′ exonuclease.

## 2. Materials and Methods

### 2.1. Materials

All synthetic oligonucleotides (ACT, HP and Fluorescein–Black hole quencher 1 (FAM– and BHQ1–labeled reporter probe), Alt–R^®^ A.s. Cas12a (Cpf1), Ultra, and Alt-R^®^ A.s. Cas12a gRNA were purchased from IDT (Coralville, IA, USA) and used without further modification (see Table S1 in Supporting Information for oligonucleotide sequence details). rCutSmart™ Buffer, Exo III, Exonuclease I (Exo I), Exonuclease T (Exo T), Lambda Exonuclease (λ Exo), and Nt.AlwI were purchased from New England BioLabs Inc. (Beverly, MA, USA). T4 Polynucleotide Kinase was purchased from Thermo Fisher Scientific (Waltham, MA, USA). Alkaline phosphatase and human serum were purchased from Sigma–Aldrich (St. Louis, MO, USA). Distilled diethyl pyrocarbonate water (DEPC-DW) and GreenStar^TM^ Nucleic Acid staining solution were purchased from Bioneer (Daejeon, Republic of Korea). Vera–tech (Hwaseong, Republic of Korea) supplied 0.5 M Ethylene diamine tetraacetic acid (EDTA) at pH 8.0.

### 2.2. Exo III Activity Detection Assay

To make a total 20 μL of Exo III reaction solution, 14 μL of DEPC–DW, 2 μL of 10×rCutSmart™ Buffer (50 mM Potassium Acetate, 20 mM Tris–Acetate, 10 mM Magnesium Acetate, 100 μg/mL Recombinant Albumin, pH 7.9), 2 μL of HP (100 nM), and 2 μL of Exo III at varying concentrations were mixed and incubated at 37 °C for 20 min to complete the specific digestion of the HP, and finally inactivated at 70 °C for 20 min. After the Exo III reaction was complete, the detection solution was prepared. Cas12a mixture consisting of 13.2 μL of DEPC–DW, 2 μL of 10× rCutSmart™ Buffer (50 mM Potassium Acetate, 20 mM Tris–Acetate, 10 mM Magnesium Acetate, 100 μg/mL Recombinant Albumin, pH 7.9), 0.4 μL of Cas12a (1 μM), 0.4 μL of gRNA (1 μM), and 4 μL of FAM– and BHQ1–labeled reporter probe (F–Q reporter) (1 μM) was added to the reaction mixture, which was then incubated at 25 °C for 30 min and inactivated at 65 °C for 15 min. The fluorescence intensity signal from the reaction solution was measured from 500 to 600 nm at an excitation wavelength of 470 nm using a Tecan M200 Pro microplate reader (Männedorf, Switzerland) and 384–well Greiner Bio–One microplates (Ref. No.781077, Courtaboeuf, France). All experiments were performed in triplicate.

### 2.3. Optimizing the Cas12a Reaction Temperature Condition of Exo III Activity Detection Assay

After the completion of the Exo III activity reaction, Cas12a mixture was prepared. Cas12a mixture consisting of 4 μL of DEPC–DW, 2 μL of 10× rCutSmart™ Buffer (50 mM Potassium Acetate, 20 mM Tris–Acetate, 10 mM Magnesium Acetate, 100 μg/mL Recombinant Albumin, pH 7.9), 2 μL of Cas12a (1 μM), 2 μL of gRNA (1 μM), and 10 μL of F–Q reporter (1 μM) was added to the reaction mixture, which was then incubated at various temperatures (20, 25, 30, 34, 37, and 45 °C) for 30 min and inactivated at 65 °C for 15 min. All experiments were performed in triplicate.

### 2.4. Optimizing the HP Loop and Stem Length Condition of Exo III Activity Detection Assay

To make a total 20 μL of Exo III reaction solution, 14 μL of DEPC–DW, 2 μL of 10× rCutSmart™ Buffer (50 mM Potassium Acetate, 20 mM Tris–Acetate, 10 mM Magnesium Acetate, 100 μg/mL Recombinant Albumin, pH 7.9), 2 μL of Exo III (5 U/mL), and 2 μL of various HP (20, 22, 24, 26, 28, 30, 32, 36bp of Stem length and 2, 4, 6, 8, and 10 bp Loop length of HP,100 nM)were mixed and incubated at 37 °C for 20 min to complete the specific digestion of the HP, and finally inactivated at 70 °C for 20 min. All experiments were performed in triplicate.

### 2.5. Polyacrylamide Gel Electrophoresis (PAGE) Analysis

To make a total 20 μL of Exo III reaction solution, 14 μL of DEPC–DW, 2 μL of 10× rCutSmart™ Buffer (50 mM Potassium Acetate, 20 mM Tris–Acetate, 10 mM Magnesium Acetate, 100 μg/mL Recombinant Albumin, pH 7.9), 2 μL of Exo III (5 U/mL), and 2 μL of HP 26 (500 nM) were mixed and incubated at 37 °C for 20 min to complete the specific digestion of the HP. The PAGE analysis procedure included separating Exo III–reacted HP 26 (500 nM), Activator (500 nM), and non–reacted HP 26 (500 nM) on a 15% polyacrylamide gel (6 mL of 30% Acrylamide, 3.6 mL of distilled water, 2.4 mL of 5X Tris–Borate–EDTA (TBE) buffer with 200 μL of ammonium persulfate (APS), and 10 μL of N,N,N’,N’–Tetramethylethylenediamine (TEMED)) under an electric field of 100 V for a duration of 60 min, with 1X TBE serving as the running buffer. After GreenStarTM Nucleic Acid staining at 30 min, the gel was scanned using the GelDoc Go Imaging System (Bio–Rad, CA, USA).

### 2.6. Exo III Inhibition Assay

To make a total 18 μL of inhibition assay solution, 12 μL of DEPC–DW, 2 μL of 10× rCutSmart™ Buffer (50 mM Potassium Acetate, 20 mM Tris–Acetate, 10 mM Magnesium Acetate, 100 μg/mL Recombinant Albumin, pH 7.9), 2 μL of Exo III (5 U/mL), and 2 μL EDTA at varying concentrations were mixed and incubated at 37 °C for 10 min before adding 2 μL of HP (100 nM). Next, the solution was incubated at 37 °C for 20 min to facilitate the digestion of the HP, and finally inactivated at 70 °C for 20 min. Cas12a mixture consisting of 13.2 μL of DEPC–DW, 2 μL of 10× rCutSmart™ Buffer (50 mM Potassium Acetate, 20 mM Tris–Acetate, 10 mM Magnesium Acetate, 100 μg/mL Recombinant Albumin, pH 7.9), 0.4 μL of Cas12a (1 μM), 0.4 μL of gRNA (1 μM), and 4 μL of F–Q reporter (1 μM) was added to the reaction mixture, which was then incubated at 25 °C for 30 min and inactivated at 65 °C for 15 min. Once all reactions are complete, fluorescence intensity signal from the reaction solution was measured from 500 to 600 nm at an excitation wavelength of 470 nm. All experiments were performed in triplicate.

## 3. Results

### 3.1. Detection Principle of Exo III Activity Assay

The overall strategy employed to determine Exo III activity using CRISPR/Cas12a is shown in Figure 1. The assay is based on the underlying principle that Exo III exclusively cleaves the 3′ end mononucleotide of dsDNA and has no effect on ssDNA. In this system, an HP acts as a substrate for Exo III. The HP stem consists of a sequence complementary to the gRNA of Cas12a; however, in the absence of a PAM sequence, collateral cleavage of the Cas12a/gRNA complex is not triggered in the loop of the dsDNA structure. Upon the introduction of Exo III, the cleavage reaction commences at the 3′ end of the HP and ends before the loop region, which comprises an ssDNA structure. Consequently, the generated single–stranded DNA probe (Activator, Act), which is complementary to the gRNA, initiates collateral cleavage of the Cas12a/gRNA complex because collateral cleavage is triggered in the case of a single–stranded DNA complementing the gRNA, even in the absence of a PAM sequence. On the contrary, when Exo III was not present, the structure of the hairpin probe (HP) remained undisturbed, and consequently, the process of collateral cleavage involving Cas12a was not initiated. This absence of Exo III activity led to the preservation of the integrity of the HP, preventing the activation of the collateral cleavage mechanism within Cas12a. As a consequence, there was no change observed in the fluorescence signals, underscoring the critical role of Exo III in driving the dynamic response of the assay.

### 3.2. Feasibility Investigation

**Figure 2:**
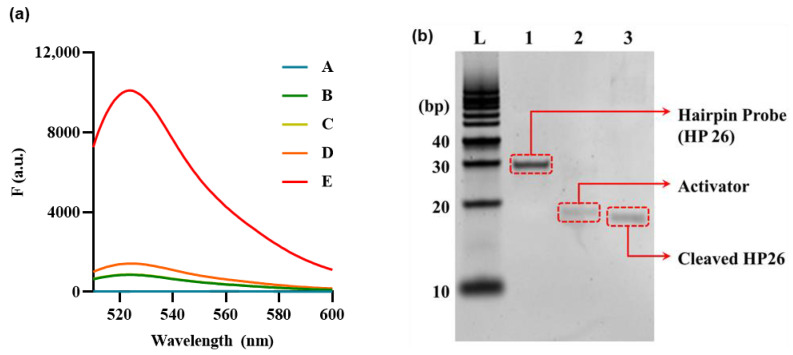
Feasibility of Exo III activity detection based on CRISPR/Cas12a collateral cleavage activity. (**a**) Fluorescence signals produced from reporter probe (F–Q) under various combinations of reaction components to verify CRISPR/Cas12a collateral cleavage activity. A: Exo III + hairpin probe (HP), B: Exo III + Cas12a/gRNA complex, C: Cas12a/gRNA complex, D: Cas12a/gRNA complex + HP, E: Exo III + Cas12a/gRNA complex + HP. The HP, Cas12a/gRNA complex, and Exo III concentrations were 5 nM, 50 nM, and 10 U/mL, respectively. (**b**) Fifteen percent polyacrylamide gel electrophoresis (PAGE) image. DNA Ladder (L). (Lane 1) HP. (Lane 2) Activator (ssDNA). (Lane 3) Cleaved HP. The concentrations of HP, Act, and Exo III were 1 uM, 1 uM, and 10 U/mL, respectively.

An experiment aimed at providing a proof–of–concept was meticulously carried out in order to establish and confirm the practical feasibility and effectiveness of the proposed approach. As shown in Figure 2a and Figure S1, in the absence of any assay components, the fluorescence signal was weak (curves A–C), and the presence of HP and Cas12a resulted in a minor background increase (Curve D). The background refers to the minimal signal in which HP is not entirely bound and reacts with Cas12a, yielding negligible intensity. However, the hydrolysis of HP with Exo III from the 3′end to the loop resulted in the release of an activator that triggered the collateral cleavage activity of Cas12a, leading to the cleavage of the F–Q reporter and the consequent generation of a remarkably significant increase in the fluorescence signal (Curve E). These findings confirm that Exo III activity accurately stimulates the collateral cleavage activity of Cas12a, thus enabling the quantification of Exo III activity in accordance with the mechanism outlined in Figure 1. The fluorescence results were further supported by polyacrylamide gel electrophoresis (PAGE) analysis of the Exo III reaction components.

PAGE analysis was performed to examine the effect of Exo III on HP. The results are presented in Figure 2b; here, Lane 1 illustrates HP 26, forming a hairpin structure that remains unresponsive to Exo III, thus maintaining its unhydrolyzed state. Lane 2 features an Activator (ACT), a 27–bp ssDNA molecule fully complementary to the gRNA sequence. In Lane 3, HP is cleaved by Exo III, positioned lower than Lane 1, which exhibited no reaction. Additionally, it can be observed at a position akin to Lane 3, displaying complete complementarity to the gRNA sequence. Based on these results, it has been confirmed that Exo III hydrolyzes HP, cleaving the stem that constitutes the dsDNA structure, thus exposing the ssDNA sequence that is complementary to the gRNA.

### 3.3. Optimization of Experimental Condition

**Figure 3:**
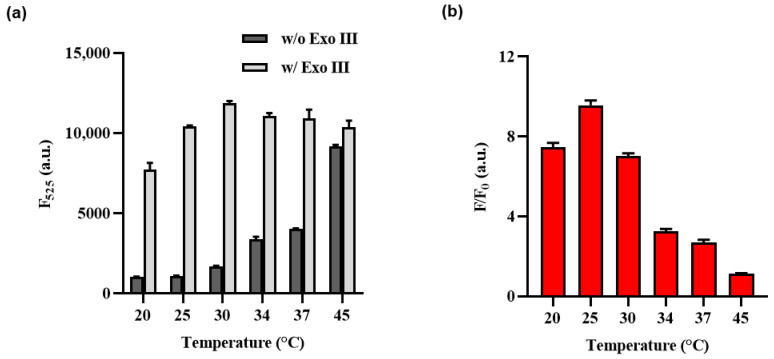
Optimization of the Exo III activity detection assay of cas12a reaction temperature. (**a**) F_525_ indicates the fluorescence intensity signal at 525 nm when Exo III is absent or present. (**b**) The fluorescence intensity ratio (F/F_0_) where F and F_0_ indicate the fluorescence intensity signal at 525 nm with and without the Exo III, respectively. The error bars indicate the standard deviations obtained from triplicate measurements.

To optimize the performance of the Exo III activity assay, various cas12a reaction temperatures and HP stem and loop length conditions were thoroughly investigated, with signal–to–background ratios (F/F_0_) used as a key parameter. As shown in Figure 3, it has been observed that elevating the reaction temperature to 30 °C or higher leads to a concurrent increase in both the fluorescence signal and background signals. These findings are consistent with the influence of temperature on the HP structure.

The increase in reaction temperature can be interpreted as leading to the instability of the HP structure at higher temperatures. Consequently, even the HP that did not react with Exo III could potentially facilitate the binding of the Cas12a/gRNA complex due to this structural instability, resulting in collateral cleavage. This interpretation aligns with the observed effects on the structural characteristics and fluorescence signal, demonstrating a correlation between the temperature–induced instability of the HP structure and its impact on both the structural features and fluorescence signal. Therefore, 25 °C was chosen as the optimal temperature for the Cas12a reaction, as it demonstrated the most significant variation in F/F_0_ ratio.

**Figure 4:**
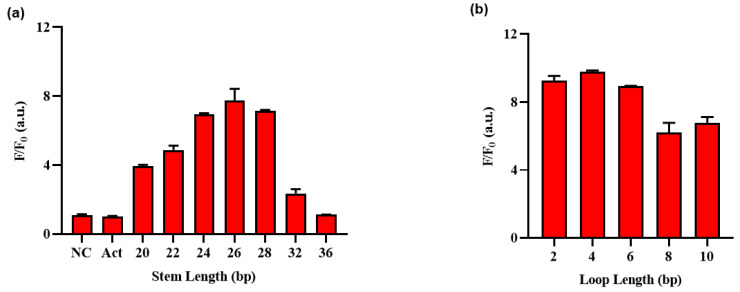
Optimization of the Exo III activity detection assay on (**a**) stem length and (**b**) loop length of HP (Table S1). F/F_0_ is the fluorescence intensity ratio where F and F_0_ indicate the fluorescence intensity at 525 nm with and without the Exo III, respectively. The error bars indicate the standard deviations obtained from triplicate measurements.

Subsequently, an optimization process was undertaken to determine the ideal lengths of the stem and loop regions within the hairpin structure of the HP. Figure 4a and Figure S2 shows that Act, a probe complementary to gRNA, produced high fluorescence intensity with or without Exo III, whereas NC, an ssDNA with no binding to gRNA, produced no fluorescence signal, regardless of the presence of Exo III. The fluorescence intensity increased with decreasing stem length, but this also led to a stronger background, indicating that shorter stem lengths resulted in an unstable hairpin structure susceptible to collateral cleavage by Cas12a at low temperatures. Therefore, HP 26 was selected as the optimal stem length and was further optimized for loop length, as shown in Figure 3b. Unlike the pattern observed in stem length, in the case of loop length, variations in length did not exert a significant impact on the background signal; thus, the four loops with the highest ratio difference were selected for sensitivity, selectivity, and EDTA inhibitory experiments with stem length 26 and loop length 4.

### 3.4. Detection Performance of Exo III Activity Assay

**Figure 5:**
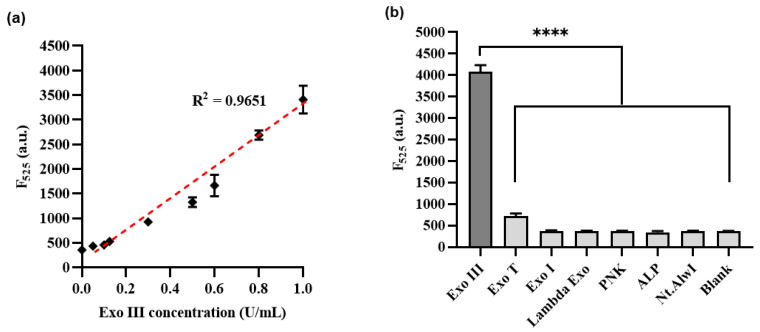
(**a**) Sensitivity of Exo III activity detection based on CRISPRs/Cas12a collateral cleavage activity. The relationship between the fluorescence intensity at 525 nm and the concentration of Exo III (U/mL). (**b**) Selectivity of the Exo III activity detection assay. The fluorescence intensity was measured in the samples containing Exo III (1 U/mL) and other proteins such as T4PNK, λ Exo, ALP, Exo T, Exo I, and Nt.AlwI (10 U/mL). The *p*–value is indicated by a star (**** means *p* ≤ 0.0001). The error bars indicate the standard deviations obtained from triplicate measurements.

To comprehensively evaluate the sensitivity of the proposed methodology, a systematic assessment was undertaken. This involved exposing Exo III to a diverse range of concentrations spanning from 0.05 to 1 U/mL, encompassing values of 0, 0.05, 0.1, 0.125, 0.3, 0.5, 0.6, 0.8, and 1 U/mL. These experiments were conducted under optimized conditions to ensure accuracy and reliability. Subsequently, the resulting fluorescence signals were meticulously recorded at a specific wavelength of 525 nm, allowing for a detailed analysis of the method’s responsiveness to varying concentrations of Exo III. Figure 5a show calibration plots of the fluorescence intensity at 525 nm in the fluorescence emission spectrum. A linear relationship was observed over the range of Exo III concentrations from 0.05 to 1 U/mL (R^2^: 0.9651), and the limit of detection (LOD) was calculated as 0.0073 U/mL, respectively, in accordance with established guidelines. The LOD was determined using the equation LOD = 3σ/S, where σ represents the standard deviation of the blank sample (σ: 7.36), and S stands for the slope of the calibration line (S: 2988.7) [34]. As shown Table 1, it becomes evident that the limit of detection for our method was comparable to or even higher than that of recently developed nanomaterial–based approaches or fluorescent signal–based methods for detecting Exo III activity.

To thoroughly assess the specificity of the developed technique, we conducted an extensive examination encompassing a range of enzymes. This set of enzymes comprised T4 polynucleotide kinase (T4PNK), lambda exonuclease (λ Exo), Exonuclease T (Exo T), alkaline phosphatase (ALP), Exonuclease I (Exo I), Exonuclease III (Exo III), and Nt.AlwI. Each of these enzymes was subjected to measurement of their respective fluorescence intensities, contributing to a comprehensive overview of the method’s selectivity. Figure 5b demonstrates a substantial increase in fluorescence intensity for Exo III, which was used at a concentration of 1 U/mL. Conversely, the other enzymes exhibited minimal fluorescence intensity signals comparable to those of the blank, despite being used at a concentration of 10 U/mL, which was more than 10 times higher than that of Exo III. These findings confirmed the remarkable selectivity of our method towards the intended activity of Exo III. Additionally, our observations validate that the meticulously designed HP configuration has demonstrated exceptional selectivity towards the activity of Exo III.

### 3.5. Inhibitory Exo III

**Figure 6:**
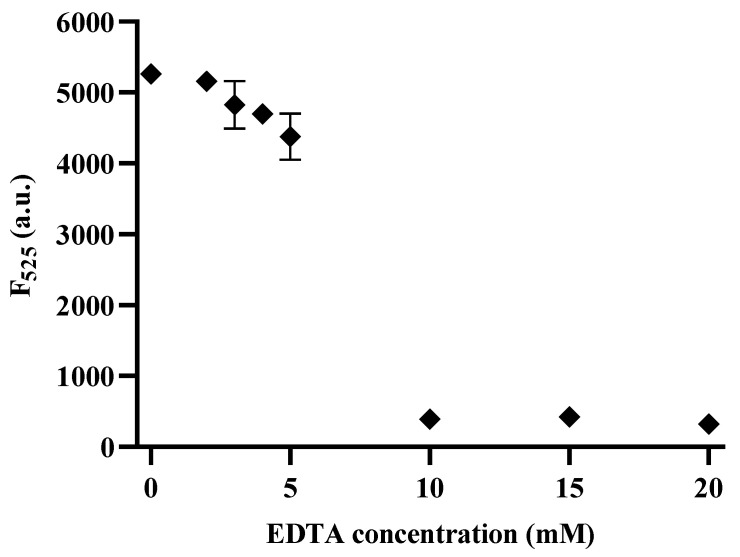
Inhibition assay of the CRISPRs/Cas12a based Exo III activity detection assay. The relationship between the Fluorescence intensity at 525 nm and the concentration of EDTA (mM). The concentration of Exo III was 5 U/mL. The error bars indicate the standard deviations obtained from triplicate measurements.

The identification of effective 3′–5′ exonuclease inhibitors is crucial for drug screening. One promising approach involves the use of EDTA, known for its chelating properties that inhibit Exo III activity [10,41]. To assess the inhibitory potential of EDTA, various concentrations ranging from 0 to 20 mM (0, 2, 3, 4, 5, 10, 15, and 20 mM) were used, and the fluorescence signals were measured at 525 nm. The results shown in Figure 6 demonstrate a clear reduction in fluorescence intensity with increasing EDTA concentrations, indicating that the chelating agent effectively inhibited Exo III activity. Notably, the half–maximal inhibitory concentration (IC_50_) of EDTA was estimated to be 6.34 mM, indicating its high potency as an Exo III inhibitor. These findings highlight the potential of our method to detect Exo III activity as a valuable tool for drug screening, particularly in the search for novel Exo III inhibitors.

### 3.6. Real–Sample Test of the CRISPR/Cas12a Based Exo III Activity Detection Assay

To confirm the robust applicability of this system for biological samples such as human serum, varying concentrations of Exo III were spiked into 5% human serum and subjected to the proposed assay. As shown in Figure S6, a linear relationship between Exo III concentration and fluorescent signal was obtained within 5% human serum. This linear relationship was observed over the range of Exo III concentrations from 0.05 to 1 unit/mL, respectively. Using this calibration curve, we successfully quantified the concentrations of Exo III in 5% human serum, achieving a coefficient of variation (CV) of less than 5.459% and a recovery ratio ranging from 102.2% to 103.5% (Table 2). In conclusion, the results highlight the capacity of the developed Exo III detection system to accurately identify Exo III in complex and heterogeneous specimens, thus establishing its robust usability for analyzing real biological samples.

## 4. Conclusions

In conclusion, our study introduces a pioneering and meticulously designed approach with remarkable sensitivity and specificity for the detection of 3′–5′ exonuclease activity through the utilization of CRISPRs/Cas12a–mediated collateral cleavage. By strategically employing a hairpin probe (HP) as an Exo III substrate, we effectively harnessed the CRISPRs/Cas12a collateral cleavage mechanism in the presence of Exo III. This strategic configuration facilitated the generation of significantly amplified fluorescence signals upon the cleavage of a neighboring reporter probe. This method exhibits high sensitivity and specificity towards Exo III, detecting activity as low as 0.0073 U/mL, which is comparable to, or even more sensitive than, other recent Exo III detection methods. Moreover, the streamlined experimental procedures and the functionality of this method in real samples enhance its practical applicability, demonstrating the versatility of this system in broader applications beyond nucleic acid detection, through the detection of enzyme activity using the CRISPRs/Cas12a system. Overall, our developed technique holds significant promise not only in fundamental research but also as a valuable tool for practical applications such as drug screening and biomolecular research.

## Figures and Tables

**Table 1:** Comparison of different methods for the determination of Exo III activity.

Method	Limit Detection (U/mL)	Linear Range (U/mL)	Ref.
Tb3+	0.8	5–100	[35]
CuNPs	0.02	0.05–2	[12]
ThT	0.5	0–10	[10]
SYBR Green I	0.7	1–200	[18]
Homo–FRET	0.17	0.25–8	[3]
luminescent	1	0–25	[36]
Graphene oxide	0.001	0.01–0.5	[37]
Photoinduced electron transfer	0.0003	0.0005–5	[38]
NMM/DAPI	4.42	10–100	[39]
Luminol	4.8	–	[40]
CRISPR/Cas12a	0.0073	0.05–1	This work

**Table 2:** Determination of Exo III in 5% human serum samples.

Added Exo III (U/mL)	Measured Exo III (μg/mL) ^a^	SD ^b^	CV (%) ^c^	Recovery (%) ^d^
0.85	0.88	188.25	4.377	103.5
0.45	0.46	123.55	5.459	102.2

^a^ Mean of three measurements. ^b^ Standard deviation. ^c^ Coefficient of variation = (standard deviation)/mean × 100. ^d^ Recovery = (measured Exo III)/(added Exo III) × 100.

## Data Availability

Not applicable.

## Supplementary Materials

The following supporting information can be downloaded at: https://www.mdpi.com/article/10.3390/bios13110963/s1, Figure S1: Feasibility of the Exo III; Figure S2: Optimization of the HP Stem and Loop length; Figures S3–S5: Optimization of the components; Figure S6: Fluorescence intensity of the 5% human serum; Table S1: Oligonucleotide sequence.
