# Supplementary material for: CRISPR/Cas12a Collateral Cleavage Activity for Sensitive 3′–5′ Exonuclease Assay
Source: Biosensors (Basel). 2023 Oct 30;13(11):963. doi: 10.3390/bios13110963 (PMC10669037; doi:10.3390/bios13110963)
Supplement: Supplementary file 1 [file biosensors-13-00963-s001.zip › biosensors-2598947-supplementary.pdf]

biosensors

MDPI

Article

CRISPR/Cas12a Collateral Cleavage Activity for Sensitive 3′–5′ Exonuclease Assay

Jae Hoon Jeung [1,2,†], Hyogu Han [1,3,†], Chang Yeol Lee [4,*], Jun Ki Ahn [1,*]

[1] Material & Component Convergence R&D Department, Korea Institute of Industrial Technology (KITECH), Ansan 15588, Korea
[2] Department of Biological Engineering, College of Engineering, Konkuk University, Seoul 05029, Korea
[3] Department of Chemistry, Gangneung–Wonju National University, Gangneung, 25457, Korea
[4] Bionanotechnology Research Center, Korea Research Institute of Bioscience and Biotechnology (KRIBB), 125 Gwahak–ro, Yuseong–gu, Daejeon, 34141, Korea

* Correspondence: lcyeol8457a@kribb.re.kr (C.Y.L); jkahn@kitech.re.kr (J.K.A)
† These authors contributed equally to this work.

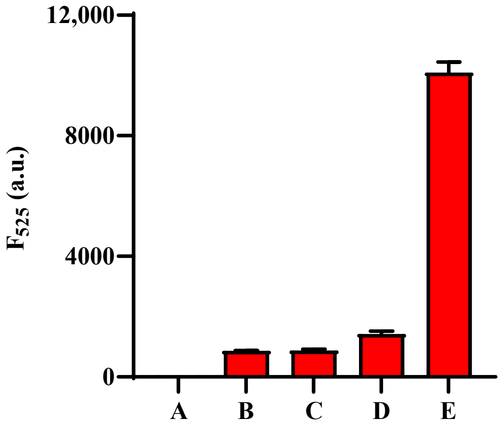

Figure S1. Feasibility of the Exo III activity assay. Fluorescence signals produced form reporter probe (F–Q) under various combinations of reaction components to verify CRISPR/Cas12a collateral cleavage activity. A: indicate Exo III + HP (hairpin probe), B: Exo III + Cas12a/gRNA complex, C: Cas12a/gRNA complex, D: Cas12a/gRNA complex + HP, E: Exo III + Cas12a/gRNA complex + HP. The HP, Cas12a/gRNA complex, and Exo III concentrations were 5 nM, 50 nM, and 10 U/mL, respectively.

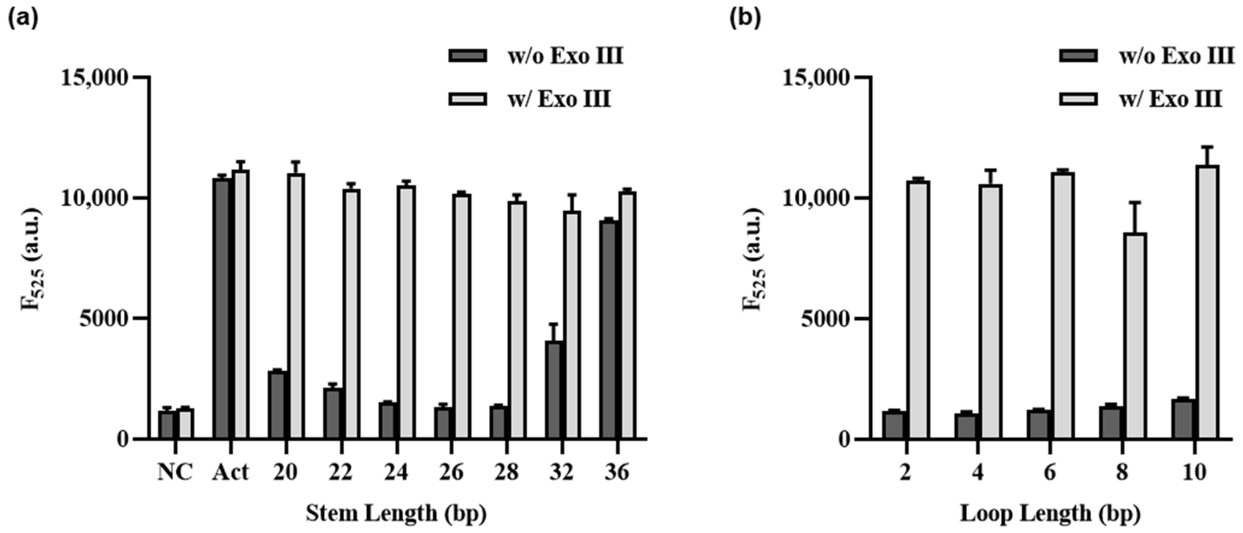

Figure S2. Optimization of the Exo III activity detection assay on (a) stem length and (b) loop length of HP (Table. S1). F_{525} indicates the fluorescence intensity signal at 525 nm when Exo III is absent or present. The error bars indicate the standard deviations obtained from triplicate measurements.

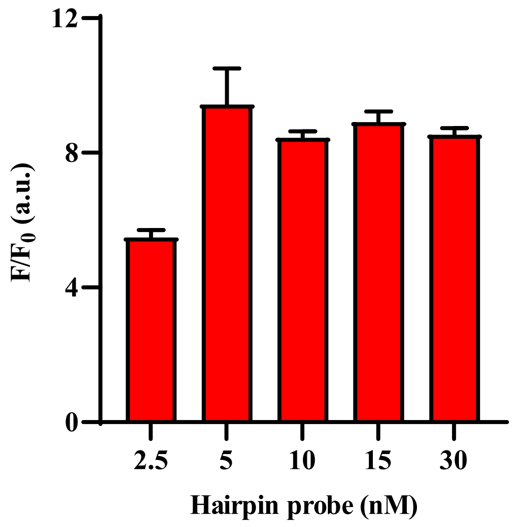

Figure S3. Optimization of the Exo III activity detection assay of HP concentration. The fluorescence intensity ratio (F/F_0) where F and F_0 indicate the fluorescence intensity signal at 525 nm with and without the Exo III, respectively. The error bars indicate the standard deviations obtained from triplicate measurements.

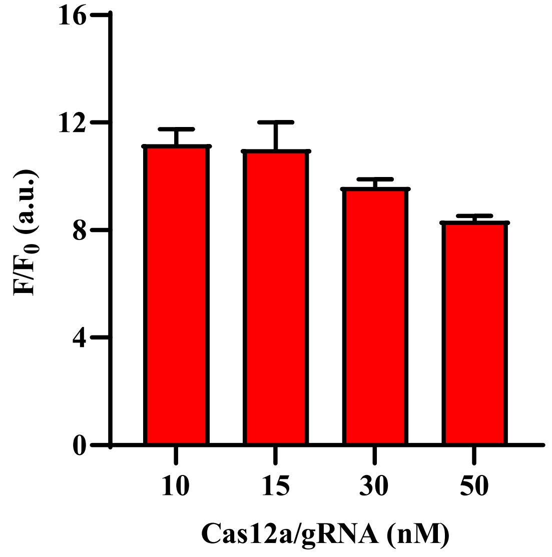

Figure S4. Optimization of the Exo III activity detection assay of Cas12a/gRNA complex concentration. The fluorescence intensity ratio (F/F_0) where F and F_0 indicate the fluorescence intensity signal at 525 nm with and without the Exo III, respectively. The error bars indicate the standard deviations obtained from triplicate measurements.

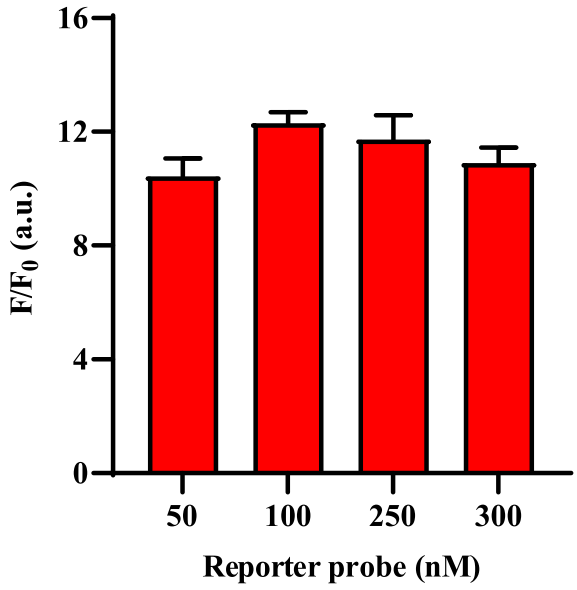

Figure S5. Optimization of the Exo III activity detection assay of reporter probe concentration. The fluorescence intensity ratio (F/F_0) where F and F_0 indicate the fluorescence intensity signal at 525 nm with and without the Exo III, respectively. The error bars indicate the standard deviations obtained from triplicate measurements.

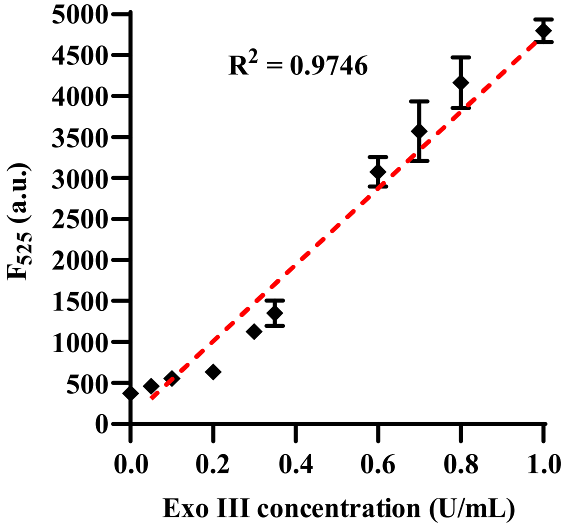

Figure S6. The Fluorescence intensity as a function of Exo III concentrations in 5% human serum. The relationship between the Fluorescence intensity at 525 nm and the concentration of Exo III (U/mL).

Table S1. Oligonucleotide sequences were used in this work.

**Oligonucleotide sequence (5′→ 3′)**	
Act	ACC TGG GGG AGT ATT GCG GAG GAA GGT
Nc	ACC TTC CTC CGC AAT ACT CCC CCA GGT
HP 20	ACC TGG GGG AGT ATT GCG GAC CCC TCC GCA ATA CTC CCC CAG GT
HP 22	GAC CTG GGG GAG TAT TGC GGA GCC CCC TCC GCA ATA CTC CCC CAG GTC
HP 24	GGA CCT GGG GGA GTA TTG CGG AGG CCC CCC TCC GCA ATA CTC CCC CAG GTC C
HP 26	GGA ACC TGG GGG AGT ATT GCG GAG GAC CCC TCC TCC GCA ATA CTC CCC CAG GTT CC
HP 28	GGA AAC CTG GGG GAG TAT TGC GGA GGA ACC CCT TCC TCC GCA ATA CTC CCC CAG GTT TCC
HP 32	GGA AGG ACC TGG GGG AGT ATT GCG GAG GAA GGC CCC CCT TCC TCC GCA ATA CTC CCC CA GG TCC TTC C
HP 36	GGA AGG TTA CCT GGG GGA GTA TTG CGG AGG AAG GTT CCC CAA CCT TCC TCC GCAA TAC TCC CCC AGG TAA CCT TCC
2 Loop	GGA ACC TGG GGG AGT ATT GCG GAG GAC CTC CTC CGC AAT ACT CCC CCA GGT TCC
6 Loop	GGA ACC TGG GGG AGT ATT GCG GAG GAC CCC CCT CCT CCG CAA TAC TCC CCC AGG TTC C
8 Loop	GGA ACC TGG GGG AGT ATT GCG GAG GAC CCC CCC CTC CTC CGC AAT ACT CCC CCA GGT TCC
10 Loop	GGA ACC TGG GGG AGT ATT GCG GAG GAC CCC CCC CCC TCC TCC GCA ATA CTC CCC CAG GTT CC
Reporter probe	(FAM) GCC AAT TGT GTG CGG AAC ACT (BHQ1)
crRNA	UrArAr UrUrUr CrUrAr CrUrCr UrUrGr UrArGr ArUrUr UrCrCr GrCrAr ArUrAr CrUrCr CrCrCr CrArGr GrUr–
